# Mast cell depletion in the preclinical phase of collagen-induced arthritis reduces clinical outcome by lowering the inflammatory cytokine profile

**DOI:** 10.1186/s13075-016-1036-8

**Published:** 2016-06-13

**Authors:** Daniël van der Velden, H. Maxime Lagraauw, Anouk Wezel, Pierre Launay, Johan Kuiper, Tom W. J. Huizinga, René E. M. Toes, Ilze Bot, Jeroen N. Stoop

**Affiliations:** Division of Biopharmaceutics, Leiden Academic Centre for Drug Research, Leiden University, Leiden, The Netherlands; Department of Rheumatology, Leiden University Medical Centre, Albinusdreef 2, 2333 ZA Leiden, The Netherlands; Université Paris Diderot, Sorbonne Paris Cité, Laboratoire d’Excellence INFLAMEX, Paris, France; INSERM U1149, Centre de Recherche sur l’Inflammation, Université Paris Diderot, Paris, France

**Keywords:** Mast cells, Collagen-induced arthritis, RMB mice, T cells

## Abstract

**Background:**

Rheumatoid arthritis (RA) is a multifactorial autoimmune disease, which is characterized by inflammation of synovial joints leading to the destruction of cartilage and bone. Infiltrating mast cells can be found within the inflamed synovial tissue, however their role in disease pathogenesis is unclear. Therefore we have studied the role of mast cells during different phases of experimental arthritis.

**Methods:**

We induced collagen-induced arthritis (CIA), the most frequently used animal model of arthritis, in an inducible mast cell knock-out mouse and determined the effect of mast cell depletion on the development and severity of arthritis.

**Results:**

Depletion of mast cells in established arthritis did not affect clinical outcome. However, depletion of mast cells during the preclinical phase resulted in a significant reduction in arthritis. This reduction coincided with a decrease in circulating CD4^+^ T cells and inflammatory monocytes but not in the collagen-specific antibody levels. Mast cell depletion resulted in reduced levels of IL-6 and IL-17 in serum. Furthermore, stimulation of splenocytes from mast cell-depleted mice with collagen type II resulted in reduced levels of IL-17 and enhanced production of IL-10.

**Conclusions:**

Here we show that mast cells contribute to the preclinical phase of CIA. Depletion of mast cells before disease onset resulted in an altered collagen-specific T cell and cytokine response. These data may suggest that mast cells play a role in the regulation of the adaptive immune response during the development of arthritis.

**Electronic supplementary material:**

The online version of this article (doi:10.1186/s13075-016-1036-8) contains supplementary material, which is available to authorized users.

## Background

Rheumatoid arthritis (RA) is characterized by progressive inflammation of the synovial joints that leads to the breakdown of cartilage and bone, eventually resulting in malformation of hands and feet, thereby reducing the quality of life for the patient [1]. In the western world, RA affects around 0.5–1 % of the general population [2]. The etiology and pathology of RA are not completely understood and environmental and genetic factors are thought to play a role in disease pathogenesis [3, 4]. Various types of immune cells, such as macrophages, B cells, T cells and mast cells have been described to contribute to the initiation and progression of joint destruction [5]. Mast cells are potent innate immune effector cells and accumulate in the synovium during RA progression. Over time, mast cells can account for up to 5 % of all nucleated cells within the inflamed synovial tissue [6, 7]. Mast cells express a wide range of surface receptors that allow them to be activated by different ligands, such as immunoglobulin E (IgE), cytokines, (endogenous) Toll-like receptor (TLR) ligands and immunoglobulin G (IgG) immune complexes [8]. Many of these ligands have been detected within the inflamed synovial tissue of RA patients. Depending on the activation route, mast cells can release a wide range of preformed mediators such as chymase, tryptase and histamine and can also release cytokines and chemokines [9]. The precise role of mast cells in the pathogenesis of RA is unknown, but activation of synovial mast cells could potentially contribute to the further progression of joint destruction either by the recruitment of leukocytes such as neutrophils and monocytes but could also facilitate the breakdown of cartilage in the joint by activating osteoclasts via release of mediators like histamine [10, 11]. To date several mouse studies have been conducted to study the role of mast cells in experimental arthritis. Different results were obtained in these studies, which could potentially have been caused by the choice of mast cell-deficient mouse strain or the method of arthritis induction [12–15]. However, most of these studies were performed in arthritis models based on the infusion of autoreactive antibodies such as with the K/BxN model. The pathogenesis of K/BxN model is based on the transfer of serum containing anti-glucose-6-phosphate (GPI) antibodies obtained from K/BxN mice. Infused anti-GPI antibodies in recipient mice will home to distal joints were they form immune complexes, which activate an inflammatory response via complement receptors, Fc receptors and is future-dependent on production of tumor necrosis factor alpha (TNF-α) and interleukin (IL)-1. Adaptive immune cells such as T cells are reported not to be required for disease induction in this model [16, 17]. Nonetheless, T cells are thought to play a major role in RA, therefore we studied mast cells in the collagen-induced arthritis (CIA) model where T cells contribute significantly to the initiation of the pathogenic immune response [18, 19]. For example, in a study conducted by Jansen et al. CD4^+^ T cells were depleted in CIA mice using either abatacept or a CD4^+^- depleting antibody. This depletion resulted in a significant reduction of collagen-specific antibodies, which coincided with a lower disease activity [20]. This study confirms the importance of T cells in the early phases of CIA in the establishment of a strong humoral immune response toward collagen type II (CII). CIA has many similarities with RA, like cartilage degradation, fibrin deposition, mononuclear infiltration, synovial cell hyperplasia, pannus formation, periosteal bone formation, and eventual ankyloses of one or more joints [18]. Comparable to human RA, CIA is composed of a preclinical (prodromal) and a clinical phase. The preclinical phase is defined as the period following the initial immunization with collagen and preceding the onset of clinical symptoms, and could be considered as a model for the prodromal stage of RA in which the underlying autoimmune response is already present but there is no visible manifestation of clinical symptoms. The clinical phase of CIA is characterized by an irreversible destruction of synovial joints. Recently, it was reported that depletion of mast cells in MCPT5-Cre-iDTR mice, before the first immunization with collagen type II (CII), could reduce the clinical outcome by altering the T cell subsets in the draining lymph nodes [21].

Although the role of mast cells in the initial onset of the pathogenic immune response is of great scientific importance, information about mast cells in later stages of disease might be more helpful for potential therapeutic intervention. Therefore the aim of this current study is to further dissect the role of mast cells in arthritis by depletion of them during these later phases of the disease.

To this end, we made use of the red mast cell basophil (RMB) mouse, which is a novel mast cell inducible knock-in mouse strain based on the transgenic expression of the simian diphtheria toxin receptor (DTR). Normally, mice are resistant to diphtheria toxin (DT) but cells who express the DTR will become highly sensitive to DT, which will cause apoptosis after challenges with DT. In the RMB mice, the DTR is expressed under control of the promoter from the β-chain of the high-affinity receptor for IgE (FcεRI) [22]. In mice, mast cells and basophils express a high-affinity receptor for IgE (FcεRI) composed of one alpha, one beta, and two gamma chains, which is essential for cell surface expression [23]. Although it has been reported that other immune cells such as dendritic cells and monocytes can express the FcεRI these cells lack the β-chain of the receptor [24, 25]. In summary, only basophils and mast cells express the β-subunit, which allows a cell-specific ablation in this current mouse model [22, 23, 26, 27]. As reported previously, basophils are depleted only for a short period of time (<12 days) and the depletion of mast cells lasts at least above 6 months in the peritoneal cavity [22]. In this study, we employed the RMB mouse to determine the contribution of mast cells to the preclinical and clinical phase of arthritis by depleting mast cells during these stages.

## Methods

### Mice

All animal work was performed in accordance with national guidelines, and experiments were approved by the animal welfare committee of the Leiden University Medical Centre.

The red mast cell and basophil mice (RMB or B6; B6.Ms4a2^tm1Mal^ mice) [22] were backcrossed for one generation with wild-type DBA/1 mice (Harlan BV, Horst, The Netherlands) in order to obtain mice that are highly susceptible for collagen-induced arthritis and in which FcεRIβ-expressing cells can be depleted by injection of DT.

### Collagen-induced arthritis and collagen antibody-induced arthritis

Collagen-induced arthritis (CIA) was induced in 8–10-week-old male RMB-DBA/1 mice by injection in the tail base with 100 μg of bovine collagen type II (CII) (2 mg/mL) (Chondrex, Inc., Redmond, WA, USA) emulsified in complete Freund’s adjuvant (CFA) (1 mg/mL; Difco, Detroit, MI, USA). On day 21 the mice received a subcutaneous boost with 100 μg CII in incomplete Freund’s adjuvant (IFA) (Difco) [28].

Collagen antibody-induced arthritis was induced by intravenous injection of 1 mg anti-collagen antibodies (Athrogen 5 Clone 2; Chondrex) intravenously on day 0 [29]. A clinical score was assigned based on a scoring protocol in which each swollen or red phalanx was given 0.5 point and 1 point per toe. A red or swollen knuckle was given 1 point, a red or swollen footpad was given 1 point and a swollen ankle and/or wrist were given 5 points. The maximum score for each paw was 15 points, resulting in a maximum possible score of 60 points per mouse. For the immunoglobulin levels, mice were bled before immunization, on day 21, and at the end of follow-up. Blood was centrifuged and serum was harvested and stored at −20 °C until use.

### In vivo depletion of FcεRIβ^+^ cells

To systemically deplete all FcεRIβ^+^ cells, mice were injected intraperitoneally (i.p*.*) for three times with a 1-day interval with 1 μg diphtheria toxin (DT) (DT unnicked, *C. diphtheria* (Cat # 322326), CalBiochem, San Diego, CA, USA), (40 ng/g bodyweight).

To deplete mast cells and basophils in the clinical phase of arthritis, mice received either DT or phosphate-buffered saline (PBS) upon clinical manifestation of arthritis. The mice were divided over two groups with a similar clinical score at the day of injection. Mast cells and basophils were depleted in one group by i.p*.* DT injection, while the control group received i.p*.* injections with PBS.

To deplete mast cells in the preclinical phase of arthritis, mice were injected with either DT or PBS starting 7 days after the first immunization. Efficiency of depletion was measured by FACS analysis for circulating basophils (CD49b^+^/FcεRI^+^/IgE^+^) 3 days after the last DT injection. At sacrifice, mast cells in the joint were visualized by staining with a napthol AS-D chloroacetate easterase staining kit (CEA) (Cat# 91C-1KT, Sigma-Aldrich, Munich, Germany). For a schematic overview of the arthritis experiment, see Additional file 1: Figure S1.

### Histology

The hind legs of arthritic mice were harvested at end of the study. Tissues were fixed in 4 % formalin and decalcified in PBS containing 10 % EDTA for 14 days before embedding into paraffin. Sections were cut 5 μm thick and either a toluene blue staining or an enzymatic staining (CEA) was performed to quantify the amount of mast cells.

To analyze the joint inflammation, sections were stained with hematoxylin and eosin (H&E). Histopathological changes were scored using the following parameters; 0: no inflammation; 1: hyperplasia of the synovial layer, infiltration of leukocytes into the joint; 2: pannus formation; 3: destruction of cartilage; and 4: destruction of bone and extensive infiltrates. The sample treatment protocol was withheld from the evaluators to prevent bias.

### Flow cytometry

At sacrifice, blood was obtained in EDTA tubes and erythrocytes were removed using a specific erythrocyte lysis buffer (0.15 M NH_4_Cl, 10 mM NaHCO_3_, 0.1 mM EDTA, pH 7.3).

Blood leukocytes were stained extracellularly to determine (a) monocytes (NK1.1^-^/Ly6G^-^/CD11b^hi^), inflammatory monocytes (NK1.1^-^/Ly6G^-^/CD11b^hi^/Ly6C^hi^/CCR2^+^), and neutrophils (NK1.1^-^/Ly6G^hi^/CD11b^hi^), (b) basophils (CD3^-^/CD4^-^/CD19^-^/CD8^-^/CD49b^+^/IgE^+^/CD117^-^), (c) T cells (CD3^+^/CD4^+^), and (d) B cells (CD19^+^/B220^+^). The antibodies used (eBioscience, Inc., San Diego, CA, USA) are summarized in Table 1. Flow cytometry analysis was performed on the FACSCanto II and data were analyzed using FACSDiva software (Becton Dickinson, Franklin Lakes, NJ, USA).

**Table 1:** Antibody panels used for flow cytometry

Staining	FITC	PE	PerCP	APC	e-Fluor-450
A	NK1.1 (Clone: PK136)	Ly6G (Clone: 1A8)	Ly6C (Clone: HK1.4)	CCR2 (Clone: 475301)	CD11b (Clone: M1/70)
B	IgE (Clone: R35-72)	CD117 (Clone: 2B8)	CD3/4/19/8 (dump channel)	CD49b (Clone: HMa2)	n/a
C	CD44 (Clone: IM7)	CCR7 (Clone: 4B12)	CD8α (Clone: 53-6.7)	CD62L (Clone: MEL-14)	CD4 (Clone: GK1.5)
D	IgM (Clone: II/41)	CD45RA (Clone: RA3-6B2)	CD19 (Clone: eBio1D3)	IgD (Clone: 11-26c)	CD5 (Clone: 53-7.3)

### Stimulation of splenocytes

At sacrifice, a single cell suspension was prepared from the spleen by using a 70-μm cell strainer (Falcon, Corning, NY, USA). Erythrocytes were removed using a specific erythrocyte lysis buffer (0.15 M NH_4_Cl, 10 mM NaHCO_3_, 0.1 mM EDTA, pH 7.3).

Regulatory T cell numbers were determined by staining extracellular with eFluor-450-conjugated rat anti-mouse CD4. Next, cells were fixed and permeabilized according to the supplier’s protocol (eBioscience). Subsequently, cells were stained with APC-conjugated rat anti-mouse/human FoxP3 or corresponding isotype as a control (eBioscience).

To determine inflammatory T_h_17 T cell phenotype in the spleen, 400,000 splenocytes/well were cultured in 96-well round-bottom plates (Greiner Bio-One, Frickenhausen, The Netherlands) and stimulated with anti-CD3 and anti-CD28 (2 μg/mL each, eBioscience) in complete IMDM, supplemented with 10 % heat-inactivated fetal calf serum, 100 u/mL penicillin/streptomycin, 2 mM L-glutamine (PAA Laboratories, Pasching, Austria), and 20 mM β-mercaptoethanol (Sigma-Aldrich). After 1 hour, brefeldin A (Sigma-Aldrich) was added up to a concentration of 10 μg/mL to inhibit secretion of the cytokines. After an additional 4 hours of incubation, cells were washed twice with FACS buffer (PBS, 1 % BSA, 2 mM EDTA) and stained for T cell surface markers.

Cells were first stained with eFluor-450-conjugated rat anti-mouse CD4. Next, cells were fixed and permeabilized according to the supplier’s protocol (eBioscience), followed by intracellular staining with PE-conjugated rat anti-mouse IL-17 or corresponding isotype as a control (eBioscience).

For the determination of the cytokine profile, splenocytes were cultured at 300,000 cells/well in triplicate and stimulated with either αCD3/28 (2 μg/mL each) or 50 μg/mL collagen type II for 96 hours, while unstimulated cells served as controls. Subsequently, the supernatant was collected for cytokine analysis.

### Serum immunoglobulin detection and cytokine levels

Total IgG1, IgG2_a_, IgG2_c_ and IgE serum levels were determined by enzyme-linked immunosorbent assay (ELISA) according to the manufacturer’s manual (Bethyl Laboratories, Montgomery, TX, USA). Collagen-specific IgG1, IgG2_a_, IgG2_c_ and IgE were determined using an in-house ELISA procedure. In short, bovine collagen was coated overnight at a concentration of 10 μg/mL in carbonate-bicarbonate buffer (pH9.6) on NUNC Maxisorp plates. Plates were washed with PBS/0.05 % Tween20, and blocked with PBS/10 % milk for 2 hours. After washing, the plates were incubated with 1/8000 diluted serum in PBS/1 % BSA/0.05 % Tween20 for 18 hours at 4C°. The different Igs were detected using an horseradish peroxidase (HRP)-conjugated goat anti-mouse Ig antibody (Southern Biotec, Birmingham, AL, USA) diluted in PBS/1 % BSA/0.05 % Tween20. HRP enzyme activity was visualized using ABTS. As a standard, serial dilutions of a pooled serum sample from mice with CIA were used. Cytokine levels were determined using a commercially available ELISA kit (BD: IL-6, TNF, interferon (IFN)-γ, IL-10) and eBioscience (IL-17A). All cytokine ELISAs were performed according to the manufacturer’s protocol.

### Statistical analysis

Data are expressed as mean ± standard error of the mean (SEM). All data presented was tested with the Shapiro-Wilk test for normal distribution. An unpaired two-tailed Student’s *t* test was used to compare normally distributed data between two groups of animals. Data of two groups with more than one variable were analyzed by two-way analysis of variance (ANOVA) followed by a Bonferroni post hoc test.

Clinical scores of mice were compared by calculating the area under the curve (AUC) of the clinical score from each mouse overtime followed by an unpaired two-tailed Student’s *t* test. Statistical analysis was performed using Prism (Graphpad Software, Inc., San Diego, CA, USA). Probability values of *p* < 0.05 were considered significant.

## Results

### FcεRIβ^+^ cell depletion in established arthritis does not reduce clinical score or delay disease progression

**Fig. 1:**
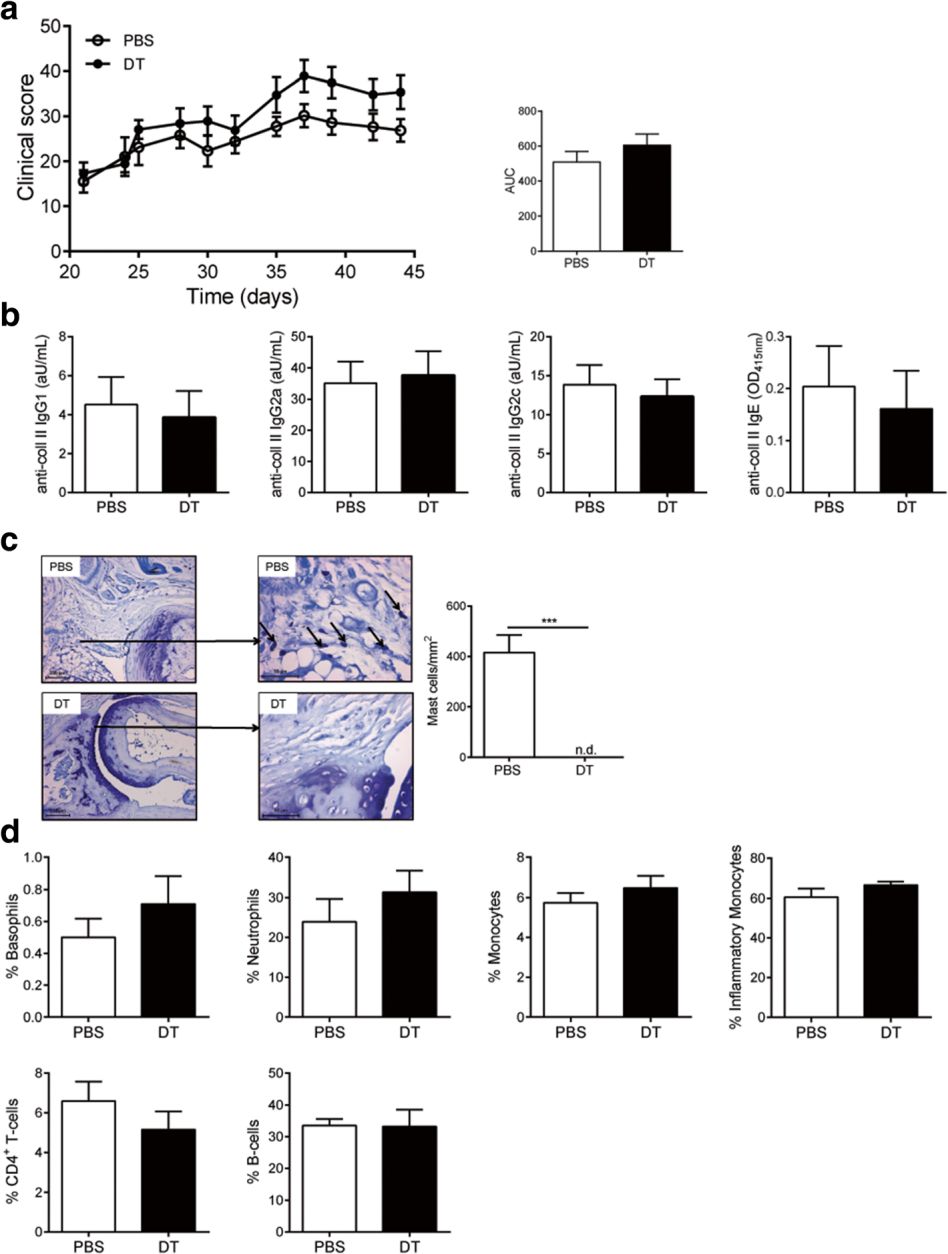
Depletion of FcεRIβ^+^-cells in clinical phase of CIA does not influence clinical outcome in RMB-DBA/1 mice. **a** Progression of CIA was monitored by clinical scoring of RMB-DBA/1 mice injected with either PBS or DT (n = 15/group). **b** Serum levels of IgG1, IgG2a, IgG2c and IgE isotype antibodies directed against collagen type II were quantified in serum from PBS- or DT-injected RMB-DBA/1 mice (n = 15/group). **c** Mast cell numbers were determined in ankle joints of PBS- and DT-treated mice (n = 15/group). *Arrows* show mast cells in the joint. **d** FACS analysis for common peripheral leucocytes in both groups (^***^ *p* < 0.001) A representative example of two independent experiments is depicted. *DT* diphtheria toxin, *IgE* immunoglobulin E, *IgG* immunoglobulin G, *PBS* phosphate-buffered saline

Arthritis is characterized by a constant activation and recruitment of immune cells into the joint leading to the destruction of cartilage and bone. Mast cells accumulate in the inflamed joint and could therefore actively contribute to the disease progression in CIA. To investigate the contribution of mast cells to the progression of established CIA in RMB-DBA/1 mice, FcεRIβ^+^ cells were depleted in the effector phase of the disease (Additional file 1: Figure S1a.). As shown in Fig. 1a, depletion of FcεRIβ^+^ cells after clinical manifestation of CIA had no effect on the clinical score. In both groups, we detected similar levels of specific immunoglobulins toward collagen type II (CII) in serum (Fig. 1b). Mast cells were present in the joints of saline-treated mice, whereas mast cell were completely absent in DT-treated mice (Fig. 1c). Further analysis of the different blood leukocyte populations by flow cytometry showed no differences in percentages of basophils, neutrophils, (inflammatory) monocytes, CD4^+^ T cells, and B cells (Fig. 1d). Taken together, these data indicate that depletion of FcεRI^+^ cells after the onset of CIA did not affect progression of CIA.

**Fig. 2:**
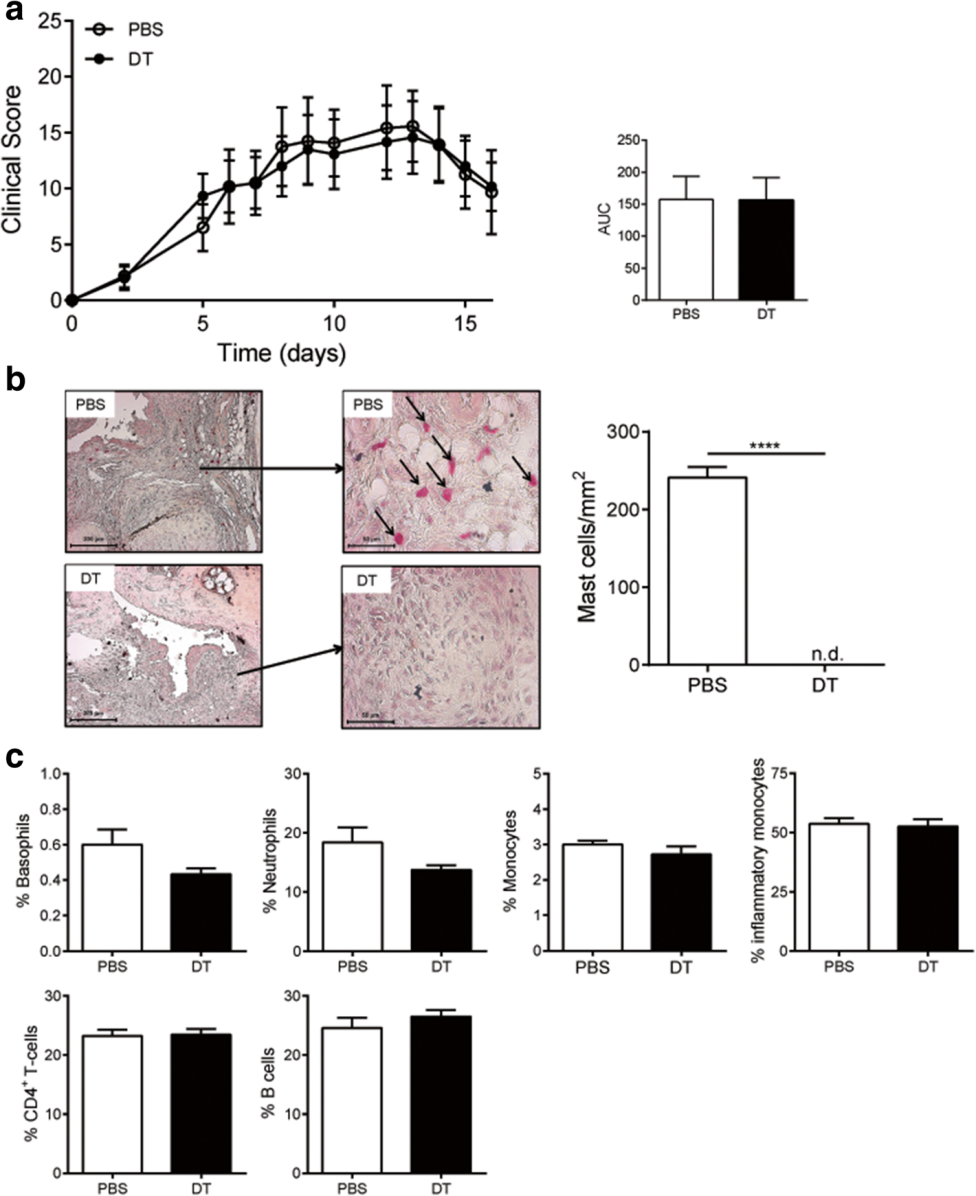
Clinical outcome of collagen antibody-induced arthritis (CAIA) is independent of FcεRIβ^+^-cells. **a** Progression of CAIA-induced arthritis in both mast cell-competent and -depleted RMB-DBA/1 mice. (n = 6/group) (**b**) Mast cell numbers in the ankle joint of PBS- and DT-injected RMB-DBA/1 mice. (n = 6/group) (**c**) FACS analysis for common peripheral leukocytes in both groups (n = 6/group). *AUC* area under the curve, *DT* diphtheria toxin, *PBS* phosphate-buffered saline

To further study the role of mast cells in the effector phase of arthritis we used the collagen antibody-induced arthritis (CAIA) model in RMB-DBA/1 mice [29]. Unlike the CIA model, this model does not require an active adaptive immune response toward collagen type II. The CAIA model depends on the injected pathogenic anti-collagen antibodies and resembles the effector phase of collagen-induced arthritis after the adaptive immune response has developed. Mast cell-deficient or -competent RMB-DBA/1 mice were intravenously (i.v.) injected with anti-collagen antibodies and scored daily for arthritis development (Additional file 1: Figure S1b). We did not observe a significant difference in clinical score between both groups (Fig. 2a). At sacrifice, a total mast cell depletion was confirmed in the joints of DT-treated mice (Fig. 2b). Flow cytometry analysis of the blood compartment showed no significant differences in blood leukocytes subsets (basophils, neutrophils, monocytes, inflammatory monocytes, T cells, and B cells) as depicted in Fig. 2c. These findings suggest that mast cells do not play a role in experimental arthritis once arthritis has fully developed and the anti-collagen type II antibody response has been developed.

### Absence of mast cells in the preclinical phase of CIA reduces clinical outcome

Mast cells can secrete various mediators that can regulate the immune response. Therefore mast cells could potentially influence the clinical course of arthritis by regulating collagen-specific B and T cell response required for the development of disease. The CII-specific response starts to develop directly after the first immunization, but mice typically do not develop arthritis until after the booster injection 3 weeks later. To investigate whether mast cells can play an immunoregulatory role during this phase of disease, we depleted FcεRIβ^+^ cells in RMB-DBA/1 mice 7 days after the first immunization as schematically shown in Additional file 1: Figure S1c.

**Fig. 3:**
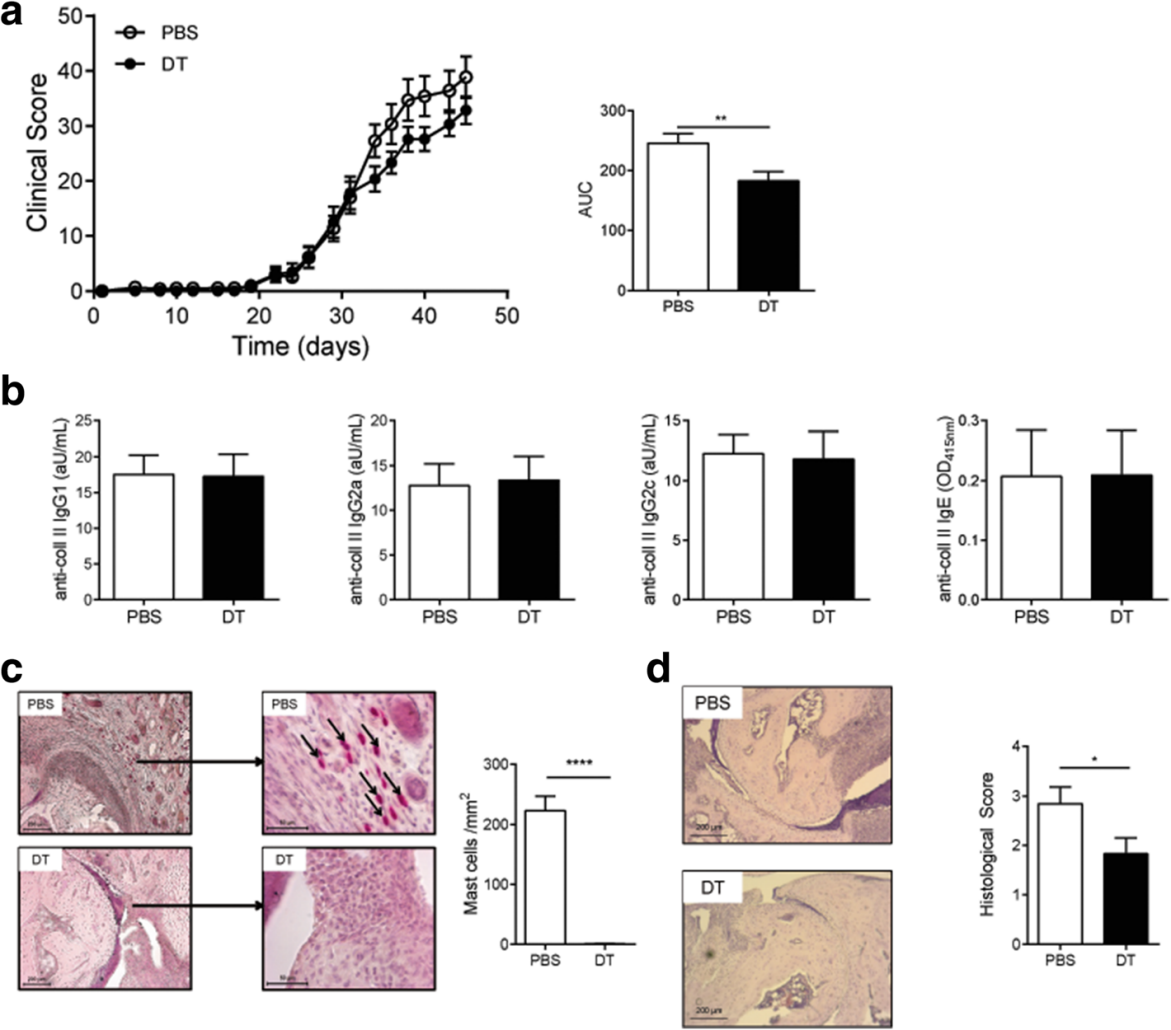
Absence of FcεRIβ^+^ cells during preclinical phase of CIA reduces clinical outcome of arthritis in effector phase of disease. **a** Progression of CIA was monitored by clinical scoring of RMB-DBA/1 mice, which have been injected with either PBS or DT in the preclinical phase of CIA (n = 15/group). **b** Serum levels of IgG1, IgG2a, IgG2c and IgE isotype antibodies directed against collagen type II were quantified in serum from PBS- or DT-injected RMB-DBA/1 mice (n = 15/group) (**c**) Mast cell numbers were determined in ankle joints of PBS- and DT-treated mice (n = 6/group). *Arrows* show mast cells in the joint. **d** Histological score of joint inflammation in the ankle joint of PBS- and DT-treated mice (n = 15/group). Representative H&E-stained sections of ankles obtained from PBS- and DT-treated RMB-DBA/1 mice. ^*^ *p* < 0.05. A representative example of two independent experiments is depicted. *AUC* area under the curve, *DT* diphtheria toxin, *IgE* immunoglobulin E, *IgG* immunoglobulin G, *PBS* phosphate-buffered saline

**Fig. 4:**
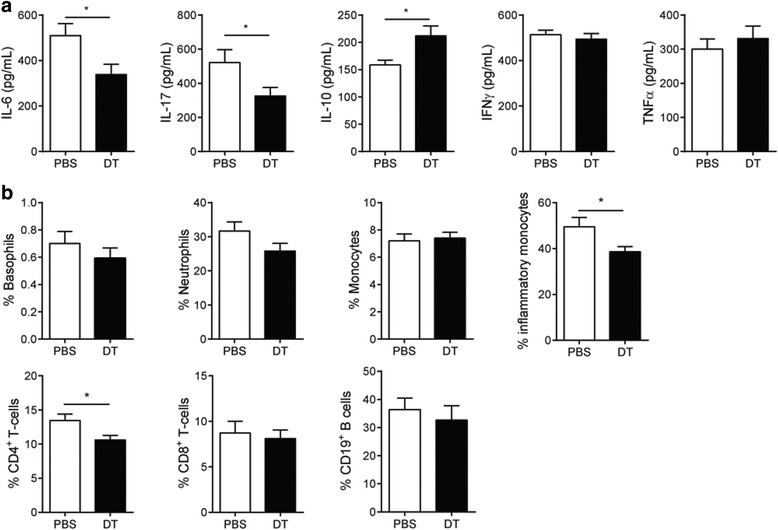
Preclinical FcεRIβ^+^ cell depletion influences systemic cytokine profile and peripheral leukocytes subsets. **a** Serum levels of IL-6, IL-17, IFN-γ, and IL-10 were quantified in serum of PBS- and DT-treated RMB-DBA/1 mice.(n = 15/group). **b** FACS analysis of the blood compartment for peripheral leukocytes (n = 15/group). (^**^ *p* < 0.01 ^***^ *p* < 0.001). A representative example of two independent experiments is depicted. *DT* diphtheria toxin, *IFN* interferon, *IL* interleukin, *PBS* phosphate-buffered saline, *TNF-α* tumor necrosis factor

Absence of FcεRIβ^+^ cells in the preclinical phase of CIA resulted in significantly lower clinical score (AUC: PBS 246 ± 16 vs. DT 183 ± 15, *p* = 0.0085) (Fig. 3a). Depletion of FcεRIβ^+^ cells did not affect anti-collagen type II antibody titers (Fig. 3b). Histological analysis showed a complete depletion of mast cells in the joints of DT-treated mice (Fig. 3c), whereas in PBS-treated mice mast cells were present in the affected joints. Further analysis of the joint inflammation in both groups showed a significant reduction in histological score in FcεRIβ^+^ cell-depleted mice (Fig. 3d). At the end of follow-up (day 45), we detected reduced serum levels of the inflammatory cytokines IL-6 (PBS 510 ± 52 vs. DT 339 ± 46 pg/mL, *p* = 0.02) and IL-17 (PBS 521 ± 75 vs. DT 326 ± 50 pg/mL, *p* = 0.04) and elevated anti-inflammatory IL-10 (PBS 158 ± 9 vs. DT 212 ± 18 pg/mL, *p* = 0.02) in the serum of FcεRIβ^+^ cell-depleted mice compared to FcεRIβ^+^ cell-competent mice (Fig. 4a). We did not observe any differences of TNF-α levels in the serum (Fig. 4a) and expression levels of TNF-α within the inguinal lymph node (data not shown). We performed flow cytometry analysis on the blood compartment for circulating basophils, monocytes, neutrophils, T cells, and B cells. Since the mice received the last DT injection more than 12 days before sacrifice, we detected a complete repopulation of basophils in DT-treated mice (Fig. 4b). Peripheral blood neutrophil and total monocytes (Fig. 4b) were not different between groups. Nonetheless we did observe a reduction of inflammatory monocytes (CD11b^+^/Ly6G^-^/Ly6C^hi^/CCR2^+^) in FcεRIβ^+^ cell-depleted mice (Fig. 4b). Furthermore, CD4^+^ T cells in FcεRIβ^+^ cell-depleted mice was decreased by 22 % (Fig. 4b), while the percentage of CD8^+^ T cells (Fig. 4b) and B cells (Fig. 4b) was not affected.

**Fig. 5:**
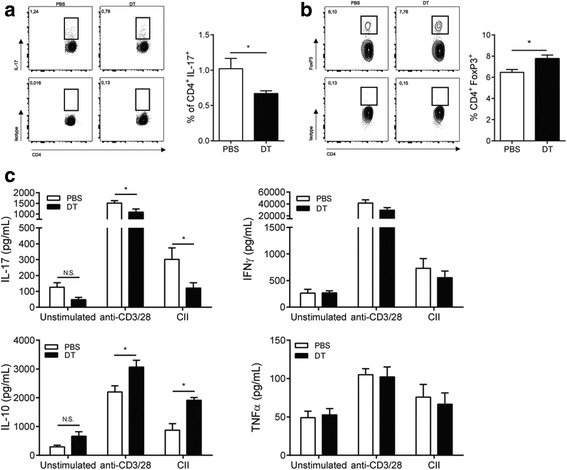
Altered CD4^+^T cell phenotype in spleen and enhanced anti-inflammatory response toward collagen type II of splenocytes from FcεRIβ^+^ cell-depleted mice. **a** Splenocytes from PBS- and DT-injected RMB-DBA/1 mice were stained intracellularly for IL-17 after stimulation with anti-CD3/28 (n = 15/group). **b** Splenocytes from PBS- and DT-injected RMB-DBA/1 mice were stained intracellularly for FoxP3. (n = 15/group). **c** Cytokine release of splenocytes from PBS- or DT-injected RMB-DBA/1 mice after restimulation with either αCD3/28 or collagen type II (n = 15/group). (^*^ *p* < 0.05). A representative example of two independent experiments is depicted. *DT* diphtheria toxin, *IFN* interferon, *IL* interleukin, *PBS* phosphate-buffered saline, *TNF-α* tumor necrosis factor

To further investigate the phenotype of the circulating CD4 T cells, we stimulated splenocytes with α-CD3/28, followed by intracellular flow cytometry staining for different T cell subsets. The balance between T_h_17 and regulatory T cells is thought to influence arthritis severity in mice [30, 31]. As shown in Fig. 5a, b depletion of FcεRIβ^+^ cells influenced T cell cytokine production, as it resulted in decreased IL-17-producing T cells (PBS 1.02 ± 0.15 vs. DT 0.67 ± 0.04 %, *p* = 0.02) and an increase in FoxP3^+^regulatory T cells (PBS 6.50 ± 0.27 vs. DT 7.80 ± 0.34 %, *p* = 0.01) (Fig. 5a, b) compared to nondepleted mice. To study the antigen-specific response toward collagen type II, splenocytes were stimulated with CII and cytokine production was analyzed by ELISA. Mast cell depletion resulted in an increase in collagen-specific IL-10 production (PBS 875 ± 225 vs. DT 1912 ± 96 pg/mL, *p* = 0.002) and a decrease in collagen-specific IL-17 production (PBS 301 ± 73 vs. DT 121 ± 33 pg/mL, *p* = 0.03) (Fig. 5c). This change in the T cell cytokine response was in line with the intracellular cytokine staining results for the CD4^+^ T cells.

As DT injections can lead to side effects, we performed additional control experiments in which wild-type mice (C57Bl/6-DBA/1) were treated with either PBS or DT in a similar experimental setup as described in Additional file 1: Figure S1c. We did not observe any effects of DT on clinical score (Additional file 2: Figure S2a), serum cytokine profile (Additional file 2: Figure S2b), and blood leukocyte subsets (Additional file 2: Figure S2c) in wild-type control mice. A specific stimulation of splenocytes resulted in an increase in IL-17 and TNF, but no differences in CII-specific changes in cytokine profile in DT-treated compared to PBS-treated wild-type mice (Additional file 2: Figure S2d). Intracellular analysis for T_h_17 and FoxP3^+^ T cells also showed no differences (Additional file 2: Figure S2e).

Taken together, these data suggests a regulatory role for mast cells in the early stages of CIA, when the immune response is established, that precedes the onset of clinical symptoms.

## Discussion

Mast cells are well known for their contribution to allergies and hypersensitivity [32]. They have also been implicated in autoimmune diseases such as RA. Increased mast cell numbers in the synovium were observed, as well as elevated levels of mast cell activation markers, such as tryptase and chymase, in synovial fluid [33].

Both in RA as well as in experimental arthritis models the possible pathogenic role of mast cells is still under debate. Several studies have been performed in mice with mutations in the gene encoding for the c-Kit receptor causing mast cell deficiency. c-Kit signaling, however, is not only essential in mast cell development and survival but also affects many other hematopoietic lineages like stem cells, innate lymphoid cells, neutrophils, and nonhematopoietic cells such as melanocytes and germ cells [34]. To circumvent the side effects of c-Kit mutations, mice have been generated with a normal Kit signaling pathway, in which mast cell deficiency is more selective. Induction of arthritis by K/BxN serum transfer in, for example, Cpa3-cre mice induced clinical arthritis, which was comparable to mast cell-competent mice [15]. Similarly, pharmacological stabilization of mast cells in the clinical phase of CIA with the drug nedocromil was unable to reduce the clinical score of DBA/1 mice compared to placebo-treated mice [35]. In contrast, it was shown that the mast cell inducible knock-out Mcpt5-cre iDTr mouse developed reduced levels of collagen-induced arthritis, when mast cells were depleted before induction of arthritis (i.e., before the first immunization with CII) [21]. This depletion reduced both the number of immune cells in the draining lymph nodes and the amount of secreted inflammatory cytokines in response to collagen II. DT treatment of these Mcpt5-Cre iDTR mice results, however, in a reduction in the number of connective tissue-type mast cells (CTMC) only, not that of mucosal mast cells (MMC) [36]. Interestingly, it is reported that mast cells in the inflamed synovium express less Mcpt5 compared to perivascular mast cells [37], indicating a microenvironmental regulation of the mast cell phenotype inside the synovium.

In the current study, we sought to investigate the contribution of mast cells to the different stages of collagen-induced arthritis, when the first immunization was done in a mast cell-competent mouse to exclude the possibility that the absence of mast cells affected the immunization efficiency. We have crossed the RMB mouse on a C57BL/6 background [22] with the DBA/1 mouse, thus generating the RMB-DBA/1 mouse, in which mast cells can be selectively depleted while being highly susceptible to the induction of CIA. We observed an >90 % incidence of CIA in these RMB-DBA/1 mice, which is comparable to the incidence in homozygous DBA/1 mice [20, 28], rendering this a valuable mouse model to study mast cells in CIA. Using this model, we were able to deplete fully FcεRIβ^+^ cells (mast cells and basophils) at any phase of disease. Activated mast cells secrete a wide range of proteases and lipid mediators, but also of a number of cytokines and chemokines, such as IL-6, IL-8 and CCL2. These cytokines are described to influence both the adaptive immune response and attraction of leukocytes to the side of inflammation [38]. Activated basophils are well known for their capacity to secrete cytokines such as IL-4, IL-13, which influence the T cell skewing toward a T_h_2 response [39]. The repopulation kinetics of mast cells and basophils after the last DT injection differs. While a complete recovery of basophils is observed within 12 days after the last DT injection, mast cells depletion lasts for the entire duration of the experiment. Therefore, it is highly likely that the majority of the observed effects in this study are due to mast cell depletion rather than the absence of basophils. Nonetheless, we cannot exclude that basophils do contribute to the immune response in this relatively short period.

Our data suggest that mast cells are involved during the initiation of arthritis and that their role is limited after the first appearance of clinical symptoms, at least in the CIA model. During the preclinical phase of CIA, we and other have detected mast cell-specific activators like collagen-specific IgE antibodies [40]. IgE-mediated activation leads to the degranulation of mast cells resulting in the release of immune-modulating mediators. Furthermore, a peak of degranulated (activated) mast cells in the knee and digits was recently shown around the booster injection in the CIA model [35]. This may suggests that mast cells contribute to the early development of an immune response in experimental arthritis. For example, it has been shown that mast cells can contribute to T cell priming through the release of TNF-α in the draining lymph nodes leading to expansion of the tissue [41]. Our ex vivo experiments indicated that mast cell depletion in the preclinical phase resulted in an altered T cell skewing, as we detected a marked reduction in IL-17 and an increase in IL-10 production by splenocytes of mast cell-depleted mice after stimulation with collagen type II.

IL-6 is a key cytokine for the development and maintenance of T_h_17 cells in mice [42]. This cytokine can be produced by various innate immune cells including mast cells. IgE-mediated mast cell activation results in high secretion of both IL-6, which is a potent promoter of Th17 cell induction, and TNF-α, which can drive the hypertrophy of the draining lymph nodes and the recruitment of naïve CD4+ T cells into the lymph node [41, 43]. In the current study, we detected collagen type II-specific IgE antibodies, indicating the presence of a CIA-specific mast cell activator. Mast cell-derived cytokines such as IL-6 may influence T cell skewing or other cellular interactions in the lymph node. The importance of IL-6 in CIA has been demonstrated by blocking IL-6, which reduces the severity of arthritis [44]. Clinical trials in human RA with anti-IL6R (tocilizumab) have demonstrated that blockade of IL-6 has therapeutic efficacy in (early) RA patients [45]. In this study, we observed lower levels of serum IL-6 in mast cell-depleted mice. This reduction of IL-6 coincided with an altered T cell skewing toward a more anti-inflammatory T cell phenotype.

T_h_17 cells have been implicated to play a role in CIA by driving arthritis progression through, e.g., osteoclast activation [44, 46]. Furthermore, it was shown that anti-IL-17 treatment significantly reduced arthritis development and severity in mice [46, 47]. Also in human RA, IL-17 and IL-17^+^ cells have been reported to contribute to RA progression. For example, elevated levels of IL-17A can be detected in serum and synovial fluid of RA patients [48], and IL-17^+^ cells can be present in synovial tissue from RA patients. Interestingly, the most abundant IL-17^+^ cell type in RA synovium were mast cells [49]. Flow cytometry analysis of stimulated splenocytes from mast cell-depleted mice showed a decrease in CD4^+^ IL-17^+^ T cells and an increase in regulatory CD4^+^ FoxP3^+^ T cells. Furthermore, we detected elevated levels of IL-10 in the supernatant of splenocytes after stimulation with collagen II. In CIA, the protective role of IL-10 has been previously been shown by both systemic treatment of IL-10 and in mice deficient for IL-10 [50–52].

Whether mast cells pay a role in the established phase of RA is not known. However, it is known that they can represent an abundant cell type in the inflamed synovium [6, 7]. Likewise, it has been shown that the autoimmune response coinciding with seropositive RA represents features of an active ongoing immune response [53]. This could also include the mast cell, which might play a role in the modulation of this response either in the inflamed synovial tissue or in the draining lymph node by the secretion of cytokines [54]. Whether mast cells also mediate other effects in RA is not known. However, the presence of mast cells in both human RA and mouse experimental arthritis suggests a contributing part. Although CIA shows many similarities with human RA, it also differs in terms of progression of arthritis. Since human RA is less progressive and shows also flares of arthritis, it could be that mast cells here do play a role.

The role of mast cells has also been investigated in other models of autoimmune diseases, such as experimental autoimmune encephalomyelitis (EAE). Similar to the results obtained from experimental arthritis studies, data from EAE studies also vary depending on the mast cell-deficient mouse strain used [55]. As circulating IL-6 and IL-17 levels were reduced in this study, it would be of interest to determine mast cell-dependent effects on EAE with our RMB mouse, since it has been shown that IL-6 and IL-17 are important in EAE development [56]. Taken together, the selective absence of mast cells can have different consequences in different diseases, depending on the time of mast cell depletion, the mouse strain used, and/or the experimental conditions used. As disease manifestation varies between individual patients, it is conceivable that the contribution of mast cells to disease development can vary between individuals, between disease stages, as well as between different diseases.

## Conclusions

In conclusion, we show that depletion of mast cells during the initiation of experimental arthritis decreases disease severity, while depletion of mast cells in established disease had no effect. Depletion of mast cells in the preclinical phase of CIA is associated with a more anti-inflammatory T cell response, suggesting that mast cells could play a role in the regulation of the adaptive immune response in early arthritis.

## Abbreviations

AUC, area under the curve; CAIA, collagen antibody-induced arthritis; CEA, chloracetate esterase; CFA, complete Freund’s adjuvant; CIA, collagen-induced arthritis; CII, collagen type II; DT, diphtheria toxin; DTR, diphtheria toxin receptor; EAE, experimental autoimmune encephalomyelitis; ELISA, enzyme-linked immunosorbent assay; FcεRI, high-affinity receptor for IgE; H&E, hematoxylin and eosin; HRP, horseradish peroxidase; IFA, incomplete Freund’s adjuvant; IFN, interferon; IgE, immunoglobulin E; IgG, immunoglobulin G; IL, interleukin; PBS, phosphate-buffered saline; RA, rheumatoid arthritis; RMB, red mast cell basophil; TNF-α, tumor necrosis factor alpha.

## Additional files

Additional file 1: Figure S1. Study outline of performed arthritis experiments in RMB-DBA/1 mice. (A) Mast cell depletion in clinical phase of CIA. (B) Collagen antibody-induced arthritis in mast cell-depleted RMB-DBA/1 mice. (C) Mast cell depletion in preclinical phase. (*DT* diphtheria toxin, *CFA* complete Freund’s adjuvant, *CII* collagen type II, *IFA* incomplete Freund’s adjuvant). (TIF 6909 kb) Additional file 2: Figure S2. DT treatment in wild-type control animals. (A) Progression of CIA was monitored by clinical scoring of C57Bl/6-DBA/1 mice injected with either PBS or DT. (B) Serum levels of IL-6, IL-17, IFN-γ and IL-10 were quantified in serum of PBS and DT-treated C57Bl/6-DBA/1 mice. (C) FACS analysis of the blood compartment for peripheral leukocytes in PBS- and DT-treated C57Bl/6-DBA/1 mice. (D) Cytokine release of splenocytes from PBS- or DT-injected C57Bl/6-DBA/1 mice after restimulation with either αCD3/28 or collagen type II, or unstimulated medium control (n = 15/group). (E) Splenocytes from PBS- and DT-injected C57Bl/6-DBA/1 mice were stained intracellularly for IL-17 after stimulation with anti-CD3/28 (n = 15/group). Splenocytes from PBS- and DT-injected C57Bl/6-DBA/1 mice were stained intracellularly for FoxP3. (^**^ *p* < 0.01, ^****^ *p* < 0.001) All graphs n = 15/group). (TIF 7185 kb)
