# Supplementary figures and images for: Mast cell depletion in the preclinical phase of collagen-induced arthritis reduces clinical outcome by lowering the inflammatory cytokine profile
Source: Arthritis Res Ther. 2016 Jun 13;18:138. doi: 10.1186/s13075-016-1036-8 (PMC4907027; doi:10.1186/s13075-016-1036-8)

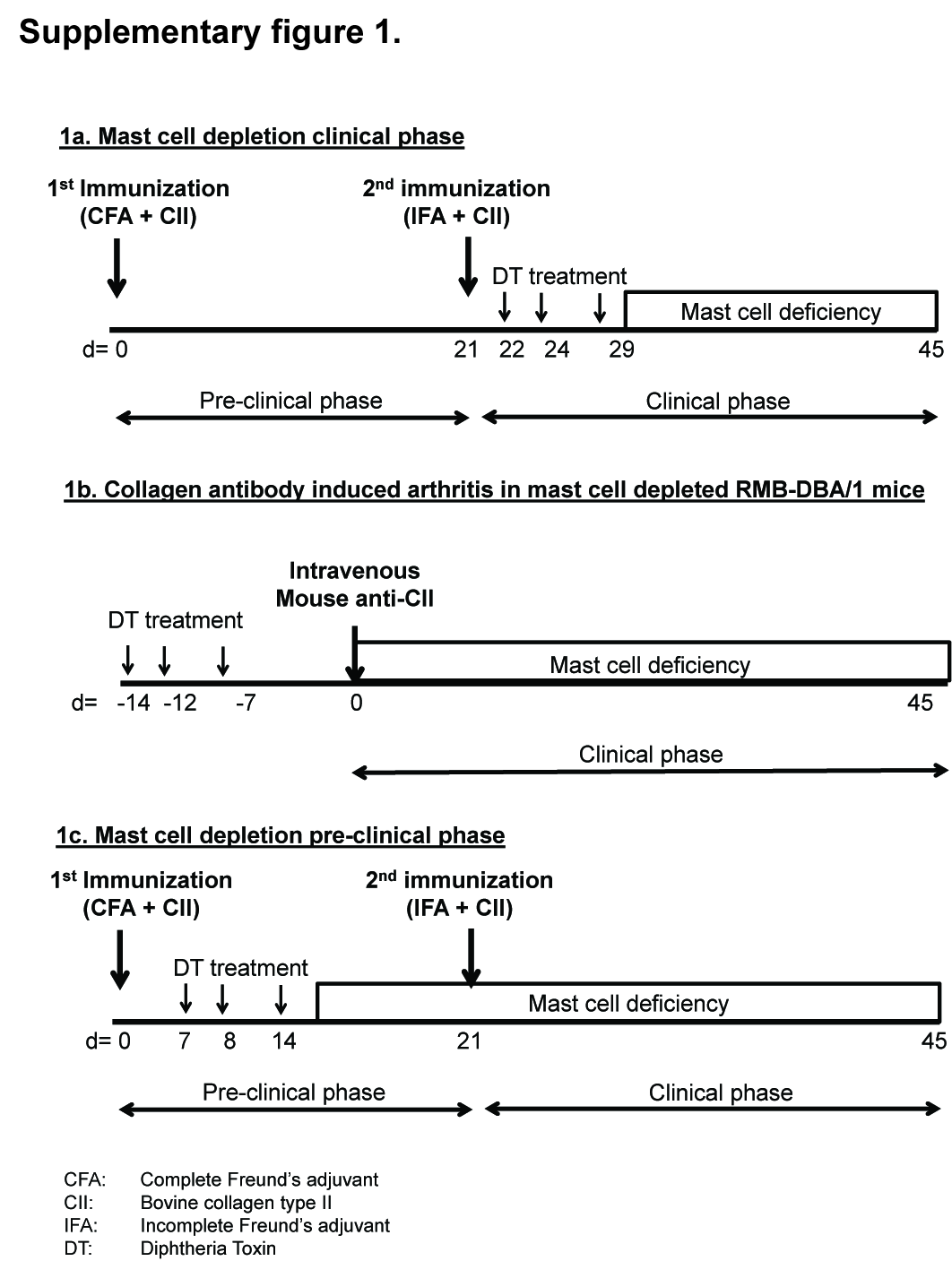

Supplement: Additional file 1: Figure S1. — Study outline of performed arthritis experiments in RMB-DBA/1 mice. (A) Mast cell depletion in clinical phase of CIA. (B) Collagen antibody-induced arthritis in mast cell-depleted RMB-DBA/1 mice. (C) Mast cell depletion in preclinical phase. (DT diphtheria toxin, CFA complete Freund’s adjuvant, CII collagen type II, IFA incomplete Freund’s adjuvant). (TIF 6909 kb) [file 13075_2016_1036_MOESM1_ESM.tif]

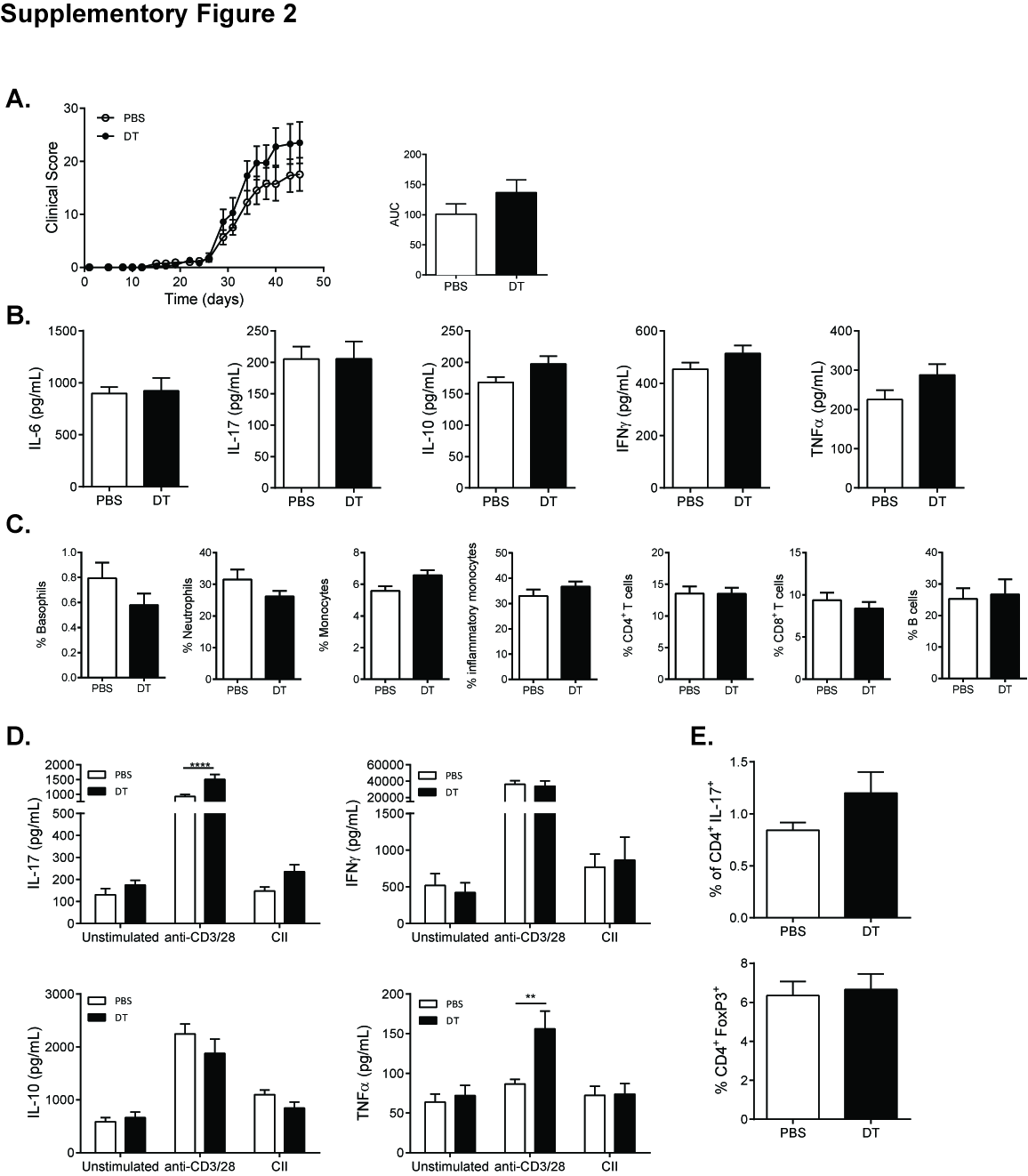

Supplement: Additional file 2: Figure S2. — DT treatment in wild-type control animals. (A) Progression of CIA was monitored by clinical scoring of C57Bl/6-DBA/1 mice injected with either PBS or DT. (B) Serum levels of IL-6, IL-17, IFN-γ and IL-10 were quantified in serum of PBS and DT-treated C57Bl/6-DBA/1 mice. (C) FACS analysis of the blood compartment for peripheral leukocytes in PBS- and DT-treated C57Bl/6-DBA/1 mice. (D) Cytokine release of splenocytes from PBS- or DT-injected C57Bl/6-DBA/1 mice after restimulation with either αCD3/28 or collagen type II, or unstimulated medium control (n = 15/group). (E) Splenocytes from PBS- and DT-injected C57Bl/6-DBA/1 mice were stained intracellularly for IL-17 after stimulation with anti-CD3/28 (n = 15/group). Splenocytes from PBS- and DT-injected C57Bl/6-DBA/1 mice were stained intracellularly for FoxP3. (** p < 0.01, **** p < 0.001) All graphs n = 15/group). (TIF 7185 kb) [file 13075_2016_1036_MOESM2_ESM.tif]
